# Interferon beta-associated recurrence of painful trigeminal neuropathy attributed to a multiple sclerosis plaque

**DOI:** 10.1186/1129-2377-15-21

**Published:** 2014-04-17

**Authors:** Antje Bischof, Till Sprenger

**Affiliations:** 1 Department of Neurology, University Hospital Basel, Petersgraben 4, CH-4031 Basel, Switzerland; 2 Department of Radiology, Division of Diagnostic and Interventional Neuroradiology, University Hospital Basel, Petersgraben 4, 4031 Basel, Switzerland

**Keywords:** Trigeminal neuralgia, Multiple sclerosis, Interferon

## Abstract

We report the case of a 49-year-old woman with painful trigeminal neuropathy in the right maxillary division attributed to a multiple sclerosis plaque as the presenting symptom of multiple sclerosis. The patient was initially treated with intravenous corticosteroids and was pain free on pregabalin for six months. She was then started on an immunomodulatory treatment and coinciding with the up-titration of interferon beta-1a, she experienced recurrence of painful trigeminal neuropathy as well as weekly migraine attacks. Worsening of primary headache disorders by interferon treatment has been previously reported. Our case suggests that treatment with interferon beta may also exacerbate symptomatic trigeminal neuralgia in multiple sclerosis.

## Background

Cranial neuralgias, most notably trigeminal neuralgia, are common symptoms in multiple sclerosis (MS) [1]. In contrast to classical trigeminal neuralgia, which is thought to be caused by neurovascular compression at the root entry zone of the trigeminal nerve, symptomatic trigeminal neuralgia (“painful trigeminal neuropathy attributed to an MS plaque” according to ICHD-3 beta nomenclature [2]) in MS typically relates to demyelinating brainstem lesions at the trigeminal entry level or the trigeminal nucleus. Worsening of primary headache disorders by interferon treatment in MS patients has been previously reported [3]. Here we describe a case suggesting that treatment with interferon beta may also exacerbate symptomatic trigeminal neuralgia in MS.

## Case presentation

We report on a 49-year-old caucasian woman presenting with excruciating paroxysmal electrical pain within the right maxillary division of the trigeminal nerve. The patient provided written informed consent to the publication of her case.

Pain occurred in a stereotyped fashion and episodes with repetitive short lasting pains lasted for up to 15 minutes without persistent pain between the paroxysms. It was triggered by touching the face or eating. No cranial autonomic symptoms were seen. She had a previous history of migraine without aura with infrequent attacks for years and a past medical history of hypercholesterolemia and lumbar discectomy. The family history was negative for headache. One sister had died from multi-organ failure probably due to long standing alcoholism.

The neurological exam revealed hypoesthesia to touch and pinprick hypoalgesia in the maxillary division of the trigeminal nerve on the right side without mechanical or thermal hyperalgesia. In addition, saccadic eye movements, incomplete internuclear ophthalmoplegia, loss of vibration in the distal lower extremities and brisk tendon reflexes were observed.

**Figure 1:**
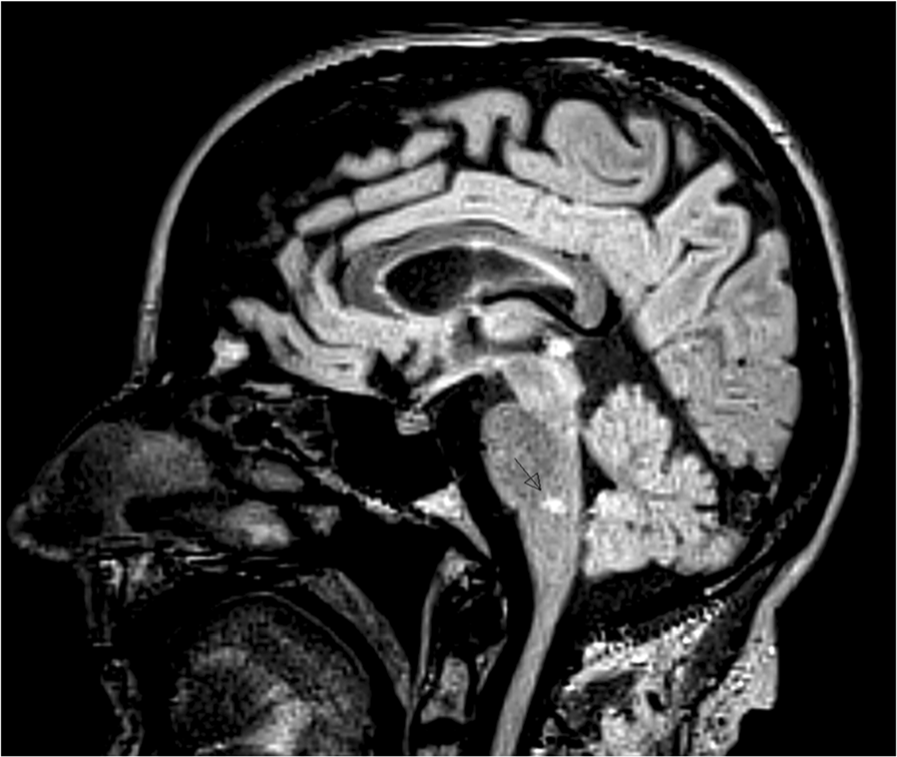
**Sagittal 3D FLAIR MR image acquired at 1.5 Tesla (Siemens Avanto) showing a hyperintense lesion in the medulla oblongata (arrow).** FLAIR = fluid attenuated inversion recovery.

Cranial and spinal MRI showed multiple periventricular, subcortical and juxtacortical T2-hyperintense lesions as well as infratentorial and spinal lesions. None of the lesions was contrast enhancing. Of note, one lesion was located in the area of the right trigeminal nucleus caudalis within the medulla oblongata (see Figure 1). There were no signs of neurovascular compression within the right trigeminal nerve root entry zone. Lumbar puncture showed five oligoclonal bands and an elevated IgG-Index with otherwise normal CSF. Visual evoked potentials were abnormal on the left.

Retrospectively, the patient reported an acute sensory loss for touch and pain one year before in the left hand and foot, which had persisted. A diagnosis of MS according to the revised McDonald criteria 2010 was made after an extensive work-up showed no evidence of alternative diagnoses [4].

After treatment with intravenous corticosteroids with oral tapering for 14 days and pregabalin 200 mg/d, the trigeminal pain improved significantly. With cessation of corticosteroids, symptoms worsened again, but with pregabalin dose increase from 200 mg/d to 500 mg/d she became pain free. Pregabalin was later slowly reduced to 250 mg/d to reduce side effects without reoccurrence of attacks for 4 months. The patient was then started on an immunomodulatory treatment for MS. She decided to start intramuscular interferon beta-1a. To minimise side effects, a four-week titration scheme was used (weekly quarter-dose increments over four weeks to full dose [30 μg]). When reaching three-quarters of the target dose, she started experiencing flu-like symptoms and occasional migraine attacks the day after the injection. At the same time, she noted a recurrence of painful trigeminal neuropathy with two attacks occurring after the injection. At full target dose, intensity and frequency of symptomatic trigeminal neuralgia strongly increased and she now always experienced migraine attacks the day after the interferon injections, whereas no migraine attacks occurred on any other days throughout the month. At this point, she was unable to brush her teeth or touch the skin without immediately evoking neuralgic pains. The pregabalin dose was augmented to 500 mg/d and combined with metamizole. After the pregabalin dose increase, the pain improved and metamizole was tapered. Seven months later, she continues to have flu-like symptoms and migraine attacks after each injection requiring high doses of non-steroidal anti-inflammatory drugs, but does not have attacks of trigeminal neuralgia while on pregabalin. Because of the temporal relationship between interferon treatment and recurrence of trigeminal neuralgia and regular migraine attacks, we discussed the possibility of switching the immunomodulatory treatment to glatiramer acetate with the patient. However, she preferred continuing the treatment with interferon, because of injection-related anxiety (injection frequency is higher with glatiramer acetate).

## Conclusions

This is to our knowledge the first detailed report of a case with an exacerbation of symptomatic trigeminal neuralgia in parallel to the onset of an immunomodulatory treatment for MS. Painful trigeminal neuropathy attributed to a multiple sclerosis plaque is defined by ICHD-3 beta criteria as pain clinically similar to classical trigeminal neuralgia [2]. Pain can be evoked by triggering stimuli such as touching or chewing. In symptomatic trigeminal neuralgia (ICHD-2), there may be sensory impairment in the corresponding division. Our patient fulfilled ICHD-2 criteria for symptomatic trigeminal neuralgia [5] and ICHD-3 criteria for painful trigeminal neuropathy attributed to a multiple sclerosis plaque [2]. The exact pathophysiological mechanisms of symptomatic trigeminal neuralgia remain unclear. In MS, demyelination of the myelinated part of the trigeminal root entry zone is a possible cause of neuralgia by ephaptic excitation of juxtaposed axons [6]. Cruccu et al. found a high lesion probability in the intrapontine trigeminal primary afferents in MS patients with symptomatic trigeminal neuralgia compared to controls and patients with trigeminal sensory disturbances [1]. Interestingly, there was no pontine lesion in our patient, but a lesion in the medullary trigeminal nucleus caudalis (Figure 1), which may have caused the trigeminal pain.

Regarding the possible effects of interferon treatment on trigeminal neuralgia in MS patients, there is only one case mentioned in the literature. This patient participated in the OWIMS study (pivotal interferon beta-1a-study) in the lower dose group and trigeminal neuralgia was reported to occur as serious adverse event, but no details of this case were published [7]. Dose dependent aggravation of primary headache, notably migraine, by interferon treatment has been repetitively described in MS patients [3]. Exact mechanisms how interferon treatment may affect headache and pain disorders are unclear. Nakatsuji et al. described an increase of interleukin 6 and TNF-alpha levels following interferon beta administration, which was associated with the development of post-injection headache and arthralgia [8]. Effects of interferon beta on cytokine levels may hence explain the worsening of migraine and the recurrence of trigeminal neuralgia in our patient.

In patients with primary headache, a comparison between interferon and glatiramer acetate treatment showed that in contrast to interferons, glatiramer acetate did not aggravate pre-existing or evoke new-onset headache [9]. To us it seems logical that the results of this study might be applicable to patients with trigeminal neuralgia, too, as our patient had a clear worsening of her symptomatic trigeminal neuralgia with the beginning of interferon beta-1a therapy, when her migraine also worsened. Whether newer MS treatments such as Teriflunomide, Dimethylfumarate or Fingolimod may impact primary or secondary headache disorders is not yet clear.

With this report we want to stress the possible clinical implications of interferon treatment on MS patients with pain syndromes, whether symptomatic or primary. Pain syndromes are a common finding in MS patients and the potential effects of immunomodulatory treatments on such headache and pain syndromes are often not sufficiently considered during immunomodulatory treatment evaluation. This is an important clinical consideration as pain may affect compliance with treatment and hence reduce the efficacy of the immunomodulation. Alternatives to interferon treatment should be considered in patients with symptomatic trigeminal neuralgia if there is new onset or recurrence of pain during treatment initiation and the pain difficult to control.

## Consent

Written informed consent was obtained from the patient for publication of this Case report. A copy of the written consent is available for review by the Editor-in-Chief of this journal.

## Abbreviations

MS: Multiple sclerosis.

## Competing interests

Antje Bischof received travel support from Biogen Idec.

Till Sprenger has consulted for Genzyme, ATI, Novartis, Eli Lilly, Allergan, Mitsubishi Pharma and Biogen Idec. He received travel support from Pfizer, Allergan, Bayer Schering and Eli Lilly.

## Authors’ contributions

Both authors equally contributed to the drafting and finalizing of this manuscript. Both authors read and approved the final manuscript.
